# Integrative Analysis of the Core Fruit Lignification Toolbox in Pear Reveals Targets for Fruit Quality Bioengineering

**DOI:** 10.3390/biom9090504

**Published:** 2019-09-18

**Authors:** Yunpeng Cao, Xiaoxu Li, Lan Jiang

**Affiliations:** 1 Key Laboratory of Cultivation and Protection for Non-Wood Forest Trees, Ministry of Education, Central South University of Forestry and Technology, Changsha 410004, Hunan, China; xfcypeng@126.com; 2 College of Life Sciences, Anhui Agricultural University, Hefei 230036, China; 3 Key Laboratory for Tobacco Gene Resources, Tobacco Research Institute, Chinese Academy of Agricultural Sciences, Qingdao 266101, China; 82101171073@caas.cn; 4 School of Economics and Law, Chaohu University, Hefei 238000, China

**Keywords:** lignin toolbox, expression, pear, phylogenetic analysis

## Abstract

Stone cell content is an important factor affecting pear fruit flavor. Lignin, a major component of pear stone cells, hinders the quality and value of commercial fruit. The completion of the Chinese white pear (*Pyrus bretschneideri*) genome sequence provides an opportunity to perform integrative analysis of the genes encoding the eleven protein families (i.e., *PAL*, *C4H*, *4CL*, *HCT*, *C3H*, *CSE*, *CCoAOMT*, *CCR*, *F5H*, *COMT,* and *CAD*) in the phenylpropanoid pathway. Here, a systematic study based on expression patterns and phylogenetic analyses was performed to identify the members of each gene family potentially involved in the lignification in the Chinese white pear. The phylogenetic analysis suggested that 35 *P. bretschneideri* genes belong to bona fide lignification clade members. Compared to other plants, some multigene families are expanded by tandem gene duplication, such as *HCT*, *C3H*, *COMT*, and *CCR*. RNA sequencing was used to study the expression patterns of the genes in different tissues, including leaf, petal, bud, sepal, ovary, stem, and fruit. Eighteen genes presented a high expression in fruit, indicating that these genes may be involved in the biosynthesis of lignin in pear fruit. Similarly to what has been observed for *Populus trichocarpa*, a bimolecular fluorescence complementation (BiFC) experiment indicated that *P. bretschneideri C3H* and *C4H* might also interact with each other to regulate monolignol biosynthesis in *P. bretschneideri*, ultimately affecting the stone cell content in pear fruits. The identification of the major genes involved in lignin biosynthesis in pear fruits provides the basis for the development of strategies to improve fruit quality.

## 1. Introduction

Lignin, an aromatic heteropolymer, is one of the major structural substances produced during the secondary thickening of plant cell walls [1,2,3,4]. The role of lignin is to provide impermeability and rigidity to plant cell walls and to protect plant tissues from pathogens. Lignin consists of different phenylpropanoids, mainly the monolignols p-coumaryl, coniferyl, and sinapyl alcohols, which differ in their degree of methoxylation [1,5]. When these monolignols are incorporated into lignin, they are referred as to p-hydroxyphenyl (H), syringyl (S) and guaiacyl (G) units, respectively [1,5]. Previous studies have shown that there are great differences in the lignin composition and content within and between species, among organs and/or tissues of the same plant, in response to environmental conditions experienced by plants, and in different developmental processes in plants [3,6,7].

Generally, the major bottleneck of the wood industry was reported to be the resistance of lignin to degradation [8,9,10]. The corrosive and expensive chemical treatments are necessary to overcome that resistance during pulp and paper manufacturing processes [9,10]. Recent studies have provided evidence that lignin is a crucial factor in determining fruit final mouthfeel, which is the most important trait of the fruit [11,12,13]. Therefore, the lignin biosynthetic pathway was always the hot point in the studies of fruits species [11,12,13]. Compared to previous studies, the lignin biosynthetic pathway has been identified to be more complex, which has been engineered several times in recent decades [1,14,15,16]. Meanwhile, several new pathways, such as CSE (caffeoyl shikimate esterase), have been discovered [17,18,19,20]. For example, together with 4CL, CSE can hydrolyze caffeoyl shikimate into caffeoyl-CoA, which bypasses the second reaction carried out by HCT. Remarkably, the previously published manuscripts showed that CSE may be important for lignification in only a few species [17,19,20]; it is not known whether this catalytic step is essential for some groups of plants—grasses and other monocots, for example. Altogether, there are eleven gene families involved in the synthesis of monolignols, namely the PAL (phenylalanine ammonia-lyase), C4H (cinnamate 4-hydroxylase), 4CL (4-coumarate:CoA ligase), HCT (shikimate O-hydroxycinnamoyl-transferase), C3H (p-coumarate 3-hydroxylase), CSE (caffeoyl shikimate esterase), CCoAOMT (caffeoyl CoA 3-O-methyltransferase), CCR (cinnamoyl CoA reductase), F5H (ferulate 5-hydroxylase), COMT (caffeate/5-hydroxyferulate O-methyl-transferase), and CAD (cinnamoyl CoA reductase) gene families. Though these genes encoding enzymes in lignin biosynthesis have been identified and characterized in some plant species, such as *Arabidopsis thaliana*, *Populus trichocarpa*, and *Eucalyptus grandis* [3,21], little is known about the lignin biosynthetic pathway in the economically important crop *P. bretschneideri*. The Chinese white pear (*P. bretschneideri*) is a native species of China, belonging to the subfamily Pomoideae of the family Rosaceae [22], and is widely cultivated in Asia. Previous studies have shown that the content of stone cells in pear fruit is one of the determinants of fruit quality [11,12,23]. Lignin is the main component of stone cells. Until recently, only a few lignin biosynthetic genes from the Chinese white pear have been cloned [11,12,23]. In recent years, researchers studying *P. bretschneideri* have focused on the identification and characterization of different families of transcription factor families and their corresponding functions [13,24,25], whereas a systematic analysis of monolignol biosynthesis has not been performed. In order to further analyze the monolignol biosynthesis pathway in *P. bretschneideri*, a comprehensive genome-wide analysis of lignin biosynthetic genes was carried out, as reported in the present study. By comparative phylogenetic analysis with other plants, eleven *P. bretschneideri* gene families and 35 genes belonging to bona fide clades were identified. Subsequent RNA sequencing data suggested that 18 genes located on the bona fide clades may participate in lignin biosynthesis of pear fruit.

## 2. Materials and Methods

### 2.1. In Silico Identification of P. bretschneideri Phenylpropanoid/Monolignol-Pathway Genes

We collected and analyzed the *A. thaliana* protein sequences for each of eleven gene families of the monolignol-pathway and downloaded the TAIR database (http://www.arabidopsis.org/). Subsequently, these *A. thaliana* protein function domains were examined using the PFAM (http://pfam.xfam.org/) [26] and InterProScan (http://www.ebi.ac.uk/interpro/) databases [27], as shown in Table 1. Then, we used the HMM model and BLASTp searches to predict the *P. bretschneideri* phenylpropanoid/monolignol-pathway genes by scanning the *P. bretschneideri* genome database with HMMER v3.2.1 software [28]. To further define the bona fide clades in *P. bretschneideri* (i.e., clades with homologs of genes that have been experimentally confirmed to have participated in lignification), we collected sequences of the investigated bona fide enzymes that contain true enzymatic biological/activity function according to previously published manuscripts [3,29]. 

### 2.2. Phylogenetic and Synteny Analyses

Similar to what have been reported by Carocha et al. (2015) [3], we obtained the sequences of bona fide enzymes from *Medicago truncatula*, *Nicotiana tabacum*, *Petroselinum crispum*, *P. trichocarpa*, *V. vinifera*, *A. thaliana*, *E. grandis,* and *O. sativa* verified to contain true biological function/enzymatic activity to determine the bona fide clades in *P. bretschneideri* (i.e., clades with homologs of genes that have been experimentally confirmed to be participated in lignification). MAFFT software was used to align the protein sequence, and alignments were improved manually [31]. The trees were built and computed in IQ-tree using the maximum likelihood method based on the best model as implemented in IQ-tree software [32]. Bootstrap-supported consensus trees were inferred from 1000 replicates, and the phylogenetic trees were reviewed using iTOL and Figtree software [33]. The GFF3 annotation, CDS, and protein file in *P. bretschneideri* were obtained from the GigaDB database. The Microsyn software and MCScanX pipeline were used to identify the synteny relationship of monolignol-pathway gene families in *P. bretschneideri* with *E*-value 10^−5^ [34,35].

### 2.3. Expression Analysis of P. bretschneideri Phenylpropanoid/Monolignol-Pathway Genes 

RNA sequencing data [36] for eight tissues from *P. bretschneideri* individuals were obtained from the public NCBI database. The accession numbers and sample details for above the RNA sequencing data are presented in the availability of data and materials section. The FASTX-toolkit software was used to remove low-quality base-calls (Q < 20) of raw reads. The absolute transcript abundance values obtained for the phenylpropanoid/monolignol-pathway genes were computed from FPKM values obtained with Hisat2 and Stringtie software [37], as described by Carocha et al. (2015) [3].

### 2.4. BiFC Assays

The coding sequences of *PbC3H1*, *PbC4H1,* and *PbC4H3* were amplified by PCR with specific primers (Table S1). To generate the BiFC (bimolecular fluorescence complementation) constructs, the coding region of *PbC3H1*—excluding its stop codon—was sub-cloned into pUC-SPYNE, and the coding regions of *PbC4H1* and *PbC4H3*—excluding their stop codons—were sub-cloned into the pUC-SPYCE vector by the GenRec Assembly Master Mix Kit (General Biosystems, Chuzhou, China). An equal OD of PbC3H1-SPYNE together with PbC4H1-SPYCE and PbC4H3-SPYCE were injected into 5-week-old *Nicotiana benthamiana* leaves separately for Agrobacterium-mediated transient expression [38]. For microscopic observation, a laser confocal microscope (Zeiss LSM700, Germany) was used to capture the fluorescence signals of the reconstituted YFP of the lower epidermal cells of leaves cut four days after injection according to previously published manuscripts [24]. 

### 2.5. Availability of Data and Materials

Pear Petal_1, Accession: SRR8119898; Pear Petal_2, Accession: SRR8119899; Pear Petal_3, Accession: SRR8119905; Pear Sepal_1, Accession: SRR8119889; Pear Sepal_2, Accession: SRR8119902; Pear Sepal_3, Accession: SRR8119903; Pear Ovary_1, Accession: SRR8119904; Pear Ovary_2, Accession: SRR8119891; Pear Ovary_3, Accession: SRR8119895; Pear stem_1, Accession: SRR8119890; Pear stem_2, Accession: SRR8119892; Pear stem_3, Accession: SRR8119907; Pear bud_1, Accession: SRR8119893; Pear bud_2, Accession: SRR8119894; Pear bud_3, Accession: SRR8119906; Pear Fruit1, Accession: SRR8119900; Pear Fruit2, Accession: SRR8119901; Pear Leaves1, Accession: SRR8119896; Pear Leaves2, Accession: SRR8119897.

## 3. Results and Discussion

### 3.1. PAL

**Figure 1:**
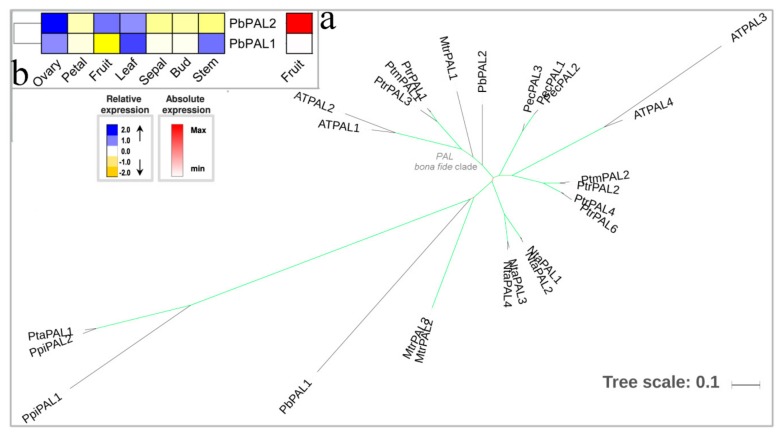
Phylogenetic tree and expression profiles of the PAL bona fide clade. (**a**) ML tree built with PAL bona fide enzymes from several species. (**b**) The expression profiles *P. bretschneideri PAL* bona fide genes. The green color of the tree branches suggests strong node support (bootstrap support ≥ 95% and SH-aLRT ≥ 75%). The FPKM values of *PbPAL* genes in different tissues are indicated in Table S3. The gene accession number is indicated in Table S4. The tree scale is the number of amino acid substitution per site.

PAL (EC: 4.3.1.5) is the first enzyme in the phenylpropanoid/monolignol-pathway that catalyzes phenylalanine deamination to cinnamic acid. Cinnamic acid is a common intermediate for the formation of plant-specific phenylpropane derivatives, a process which has been well established in many plants, including *A. thaliana* and *Populus trichocarpa* [39,40,41]. The ML tree was constructed, and then the bona fide PAL enzymes were highlighted (Figure 1a, Table S2). In comparison to *Medicago truncatula*, *Nicotiana tabacum*, *Petroselinum crispum*, and *A. thaliana*, where PAL is encoded by three or four genes, the *P. bretschneideri* only contained two PAL family members. Among them, only one PbPAL2 protein were clustered in the PAL bona fide clade, and the remaining PbPAL1 was positioned outside, which exhibited a closer evolutionary relationship with gymnosperm PAL family members. The *PbPAL1* gene presented strong expression in leaf and stem with weak expression in fruit, while the *PbPAL2* gene showed high expression in ovary and fruit (Figure 1b). Additionally, the *PbPAL2* gene was close to *AtPAL1* and *AtPAL2* in terms of its evolutionary relationship and has been reported to be mainly involved in the phenylpropanoid pathway [42,43]. By combining evolutionary relationships with expression patterns, *PbPAL2* was the most likely gene involved in the *P. bretschneideri* phenylpropanoid/monolignol-pathway.

### 3.2. The Hydroxylation Steps

C3H (EC: 1.14.14.1), C4H (EC: 1.14.11.11), and F5H (EC: 1.14.13), which belong to the cytochrome P450 family, are mainly involved in catalyzing the hydroxylation steps of the monolignol pathway [44]. 

**Figure 2:**
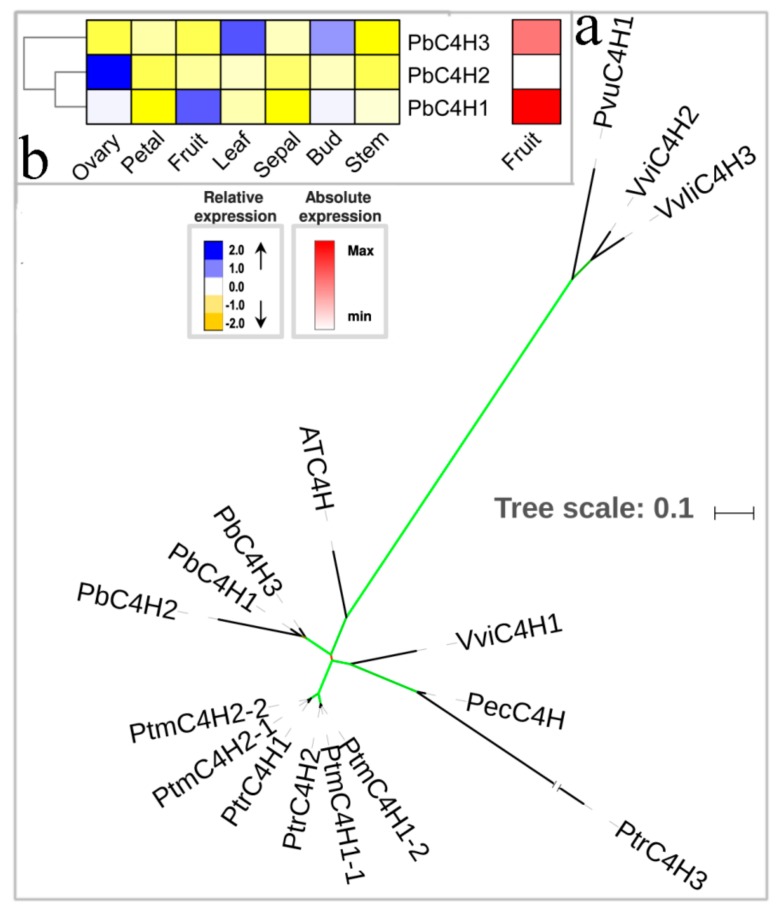
Phylogenetic tree and expression profiles of the C4H bona fide clade. (**a**) ML tree built with C4H bona fide enzymes from several species. (**b**) The expression profiles *P. bretschneideri C4H* bona fide genes. The green color of the tree branches suggests strong node support (bootstrap support ≥ 95% and SH-aLRT ≥ 75%). The FPKM values of *PbC4H* genes in different tissues are indicated in Table S3. The gene accession number is indicated in Table S4. The tree scale is the number of amino acid substitution per site.

The hydroxylation of coumaric acid to p-coumaric acid is catalyzed by C4H, the second enzyme of the phenylpropane pathway [45]. Thus far, only a single *C4H* gene has been identified in *A. thaliana*. Though multiple family members are identified in other plant species, they generally do not exceed four members. For example, *P. trichocarpa* has three *C4H* genes, and *O. sativa* contains four members. The *P. bretschneideri* genome contains three *C4H* genes encoding PbC4H1, PbC4H2, and PbC4H3 (Figure 2a, Table S2). Previously published manuscripts have confirmed that members of Class I are mainly involved in lignin biosynthesis [2,46]. In the present study, three members (*PbC4H1*, *PbC4H2,* and *PbC4H*3) were identified as Class I. *PbC4H2* was strongly expressed in the ovary, showing weak expression in other six tissues, including petal, leaf, sepal, bud, stem, and fruit. Both *PbC4H1* and *PbC4H3* showed high expression in fruit, but the *PbC4H3* gene preferentially expressed in leaf and bud. Remarkably, we also noted that the expression of *PbC4H1* was, in fruit, higher than that of *PbC4H3* (three-fold higher) (Figure 2b). Taken together, our data indicate that *PbC4H1* was the main *C4H* gene that contributed to the lignin biosynthesis of pear fruit; however, it is also very likely that the role of *PbC4H3* was not very prominent.

**Figure 3:**
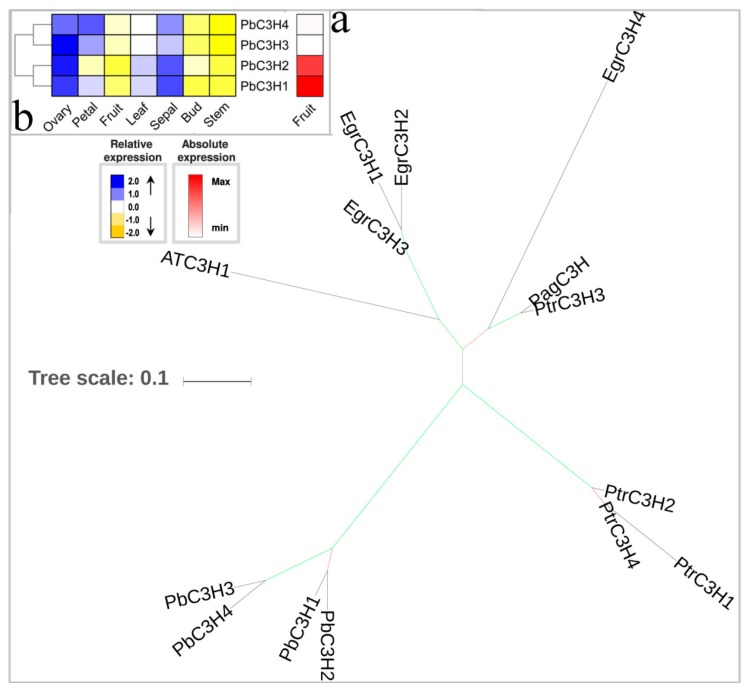
Phylogenetic tree and expression profiles of the C3H bona fide clade. (**a**) ML tree built with C3H bona fide enzymes from several species. (**b**) The expression profiles *P. bretschneideri C3H* bona fide genes. The green color of the tree branches suggests strong node support (bootstrap support ≥ 95% and SH-aLRT ≥ 75%). The FPKM values of *PbC3H* genes in different tissues are indicated in Table S3. The gene accession number is indicated in Table S4. The tree scale is the number of amino acid substitution per site.

3-hydroxylation of 4-coumaroyl-shikimate is mainly is mainly catalyzed by C3H [47]. *A. thaliana* exhibited lignin depletion in meta-hydroxylated G and S units after knocking down *C3H* [48,49]. The C3H catalyzed reaction is irreversible, mainly towards G and S lignin [49]. Though *A. thaliana* and *Oryza sativa* have single copy *C3H* genes [21], the *P. bretschneideri* genome contained four members. *PbC3H1*/*PbC3H2* and *PbC3H3*/*PbC3H4* were located in chromosome 8 and chromosome 15 (Table S2), respectively, and have been shown to be expanded by lineage-specific tandem duplications, which is consistent with this gene family in *P. trichocarpa* and *E. grandis*. The expression patterns of these four *C3H* genes were very similar and were highly expressed in the ovary and sepal and weakly expressed in bud and stem, indicating functional redundancy occurred after tandem duplication. Additionally, we also noticed that *PbC3H1* and *PbC3H2* were highly expressed in fruit compared to other two genes (2.5-fold higher) (Figure 3b). Taken together, our data indicate that both *PbC3H1* and *PbC3H2* might contribute to lignin biosynthesis of pear fruit.

**Figure 4:**
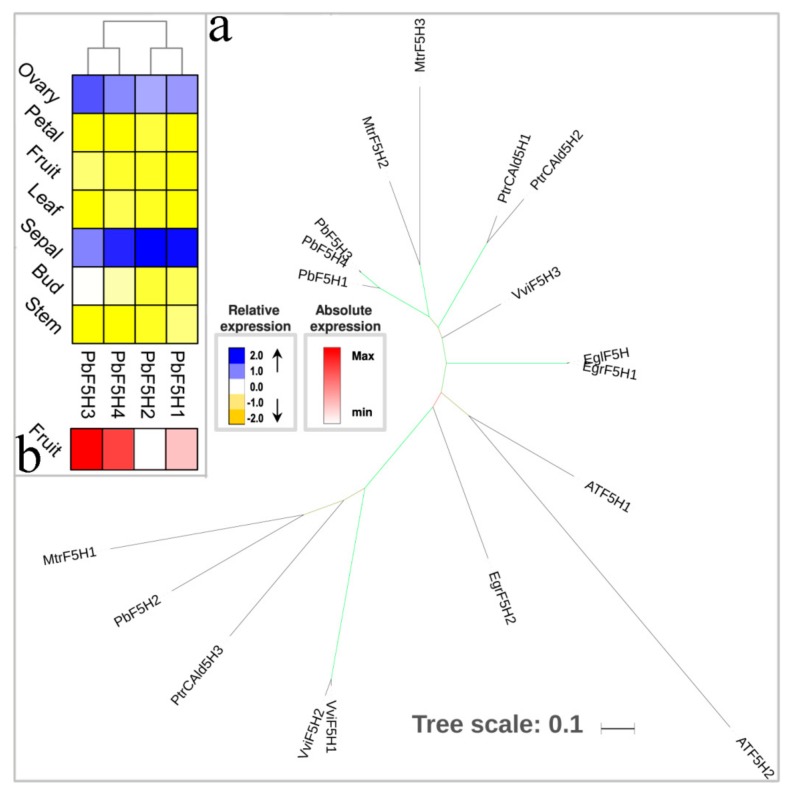
Phylogenetic tree and expression profiles of the F5H bona fide clade. (**a**) ML tree built with F5H bona fide enzymes from several species. (**b**) The expression profiles *P. bretschneideri F5H* bona fide genes. The green color of the tree branches suggests strong node support (bootstrap support ≥ 95% and SH-aLRT ≥ 75%). The FPKM values of *PbF5H* genes in different tissues are indicated in Table S3. The gene accession number is indicated in Table S4. The tree scale is the number of amino acid substitution per site.

F5H contributes to the pathway leading to sinapyl alcohol and, finally, to S lignin [14,50,51]. In *A. thaliana*, two F5H paralogs (*AtF5H1* and *AtF5H*2) were identified, and *AtF5H1* had been proved to have contributed to lignification [52,53]. Additionally, the overexpression of *F5H* showed a substantially higher proportion of S units than normal: Up to *c.* 84% in *Nicotiana tabacum*, up to 93.5% in *P. trichocarpa*, and as high as 92% in AtF5H1-up-regulated *A. thaliana* [52,54,55]. The *P. bretschneideri* genome harbors four *F5H* genes encoding PbF5H1, PbF5H2, PbF5H3, and PbF5H4 (Figure 4a). *PbF5H1* and *PbF5H2* were located in chromosome 2 and chromosome 10, respectively. Both *PbF5H3* and *PbF5H4* were placed in same chromosome (Chr 15) (Table S2). Previous studies have shown that the G lignin is greater than S lignin in pear fruit [11,12], which may be related to the low expression of *PbF5H* genes in pear fruit. To further identify the *PbF5H* genes that might be involved in lignin synthesis in pear fruits, we performed the absolute expression level of *PbF5H* genes in pear fruits. Compared to *PbF5H2* gene, *PbF5H1*, *PbF5H3* and *PbF5H4* were relatively highly expressed in fruit. These three genes are therefore probably involved in lignin biosynthesis of pear fruit.

### 3.3. 4CL

**Figure 5:**
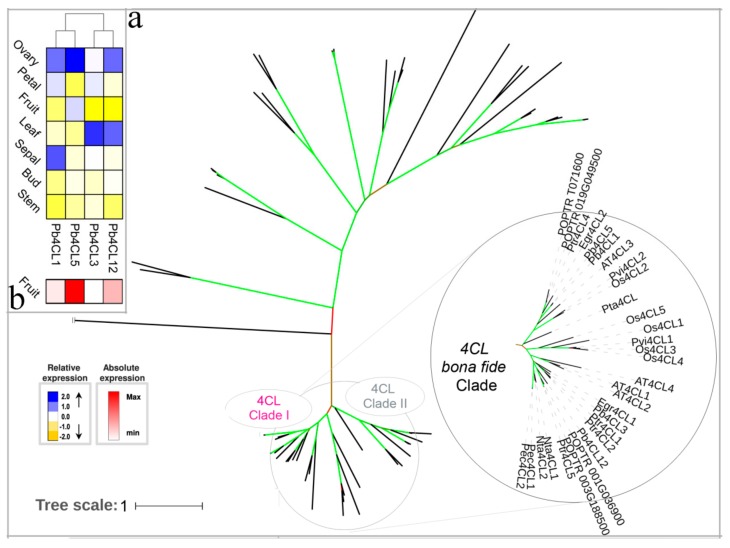
Phylogenetic tree and expression profiles of the 4CL bona fide clade. (**a**) ML tree built with 4CL bona fide enzymes from several species. (**b**) The expression profiles *P. bretschneideri 4CL* bona fide genes. Subclade containing the bona fide genes is highlighted. The green color of the tree branches suggests strong node support (bootstrap support ≥ 95% and SH-aLRT ≥ 75%). The FPKM values of *Pb4CL* genes in different tissues are indicated in Table S3. The gene accession number is indicated in Table S4, Figure S1. The tree scale is the number of amino acid substitution per site.

4CL is located at the center of the pathway and is a key step in the metabolism of phenylpropanoid [56]. Many *4CL* members have been identified and biochemically characterized in *A. thaliana* and *P. trichocarpa* [56,57]. The *4CL* gene family contain the ACS (acyl: CoA synthetase) superfamily, allowing for discrimination of the 4CL bona fide clade, clustered into two clades based on previously published manuscripts [21,58]. Unlike *A. thaliana*, *E. grandis,* and *P. trichocarpa*, *P. bretschneideri* has two *4CL* genes (*Pb4CL1* and *Pb4CL5*) in clade II related to soluble phenolic and flavonoid biosynthesis (Figure 5a). The *P. bretschneideri* genome had two members (*Pb4CL3* and *Pb4CL12*) in clade I, which is consistent with the dicots with two-to-four members in this clade. The *Pb4CL3* and *Pb4CL12* genes were highly expressed in the leaf, followed by the ovary. Compared to the *Pb4CL3* gene, *Pb4CL12* was highly expressed in fruit (Figure 5b). By combining the roles of a clade I members in lignin biosynthesis and expression analysis, the strong expression of *Pb4CL12* in fruits may involve the lignin biosynthesis of pear fruit.

### 3.4. HCT

**Figure 6:**
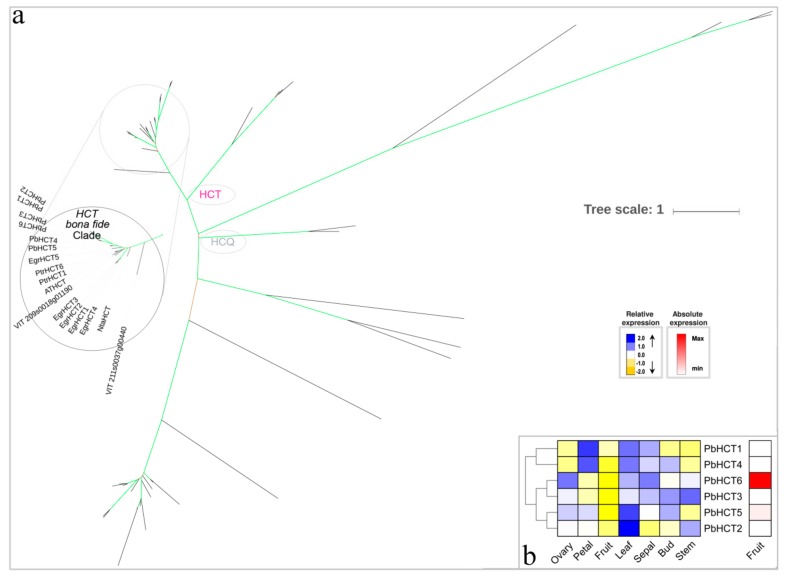
Phylogenetic tree and expression profiles of the HCT bona fide clade. (**a**) ML tree built with HCT bona fide enzymes from several species. (**b**) The expression profiles *P. bretschneideri HCT* bona fide genes. Subclade containing the bona fide genes is highlighted. The green color of the tree branches suggests strong node support (bootstrap support ≥ 95% and SH-aLRT ≥ 75%). The FPKM values of *PbHCT* genes in different tissues are indicated in Table S3. The gene accession number is indicated in Table S4, Figure S2. The tree scale is the number of amino acid substitution per site.

HCT can catalyze C3H to produce substrates for the 3-hydroxylation of hydroxy cinnamyl phenolic rings, with the reactions both immediately preceding and following the insertion into monolignol precursors [59,60]. With caffeyl-CoA and p-coumaroyl-CoA as the preferred substrates, HCT is capable of transferring an acyl group to the acceptor compound shikimic acid, which ultimately produces p-coumaroyl shikimate [59,60]. Subsequently, we found that six *PbHCT* genes were identified corresponding to the bona fide HCT (Figure 6a). These *PbHCT* genes exhibited a lineage-specific expansion as compared with the other dicots, such as *A. thaliana* and *M. truncatula*, which only contained one HCT gene each; *P. trichocarpa* had two genes. *PbHCT1*/*PbHCT2* and *PbHCT5*/*PbHCT6* were in a tandem arrangement of chromosome 4 and chromosome 17, respectively, suggesting that these gene pairs have undergone tandem gene duplication events (Table S2). Both *PbHCT1*/*PbHCT2* and *PbHCT5*/*PbHCT6* exhibited a very distinct profile; for instance, *PbHCT1* was highly expressed in the petal, and *PbHCT2* was preferentially expressed leaves, indicating that functional divergence occurred after tandem duplication events (Figure 6b) [22]. Additionally, *PbHCT6* was highly expressed in fruit compared to other genes (40-fold higher), so this gene was probably involved in lignin biosynthesis of pear fruit.

### 3.5. CSE

**Figure 7:**
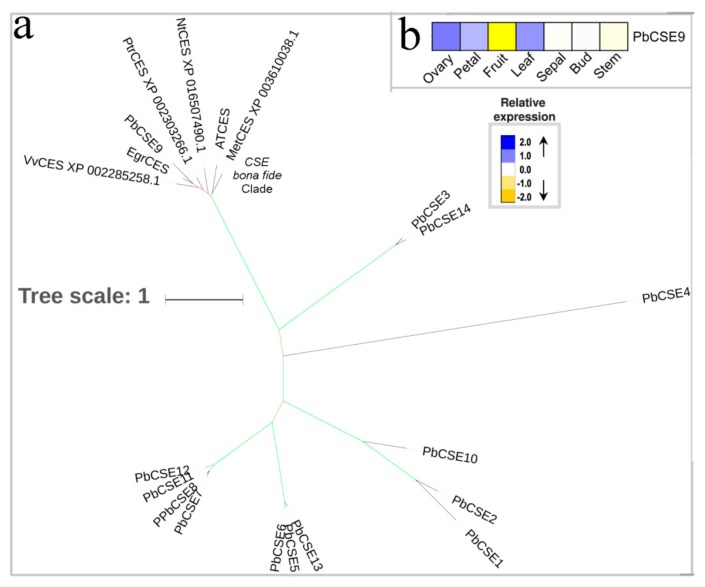
Phylogenetic tree and expression profiles of the CSE bona fide clade. (**a**) ML tree built with CSE bona fide enzymes from several species. (**b**) The expression profiles *P. bretschneideri CSE* bona fide genes. The green color of the tree branches suggests strong node support (bootstrap support ≥ 95% and SH-aLRT ≥ 75%). The FPKM values of *PbCSE* genes in different tissues are indicated in Table S3. The tree scale is the number of amino acid substitution per site.

The CSE enzyme has been identified to be involved in the *A. thaliana* lignin biosynthetic pathway. Its main role is to catalyze the produced caffeic acid [17]. Compared with wild-type *A. thaliana*, the CSE mutant plants showed a reduced lignin content and an increased relative proportion of p-hydroxyphenyl in the lignin polymer [17]. CSE can form an alternative pathway to caffeyl-CoA with 4CL [17]. In the present study, we obtained its orthologous *CSE* genes from the NCBI database by using the *ATCSE* gene that has been previously characterized. Ha et al. (2016) have shown that *MetCSE* plays an essential role for lignification in *M. truncatula* [19]. Subsequently, we generated the *CSE* phylogenetic tree and found that the *PbCSE9* gene belongs to the bona fide CSE enzymes (Figure 7a). The expression pattern of *PbCSE9* suggested that it expressed in all seven tissues tested (Figure 7b) and preferentially and strongly expressed in the ovary and leaf but weakly expressed in the fruit. Remarkably, the *PbCSE9* has a closer evolutionary relationship to the *E. grandis*, *M. truncatula*, *A. thaliana,* and the *V. vinifera CSE* genes (Figure 7a). Recent studies have shown that the *CSE* genes of *E. grandis*, *M. truncatula* and *A. thaliana* are involved in lignification [17,19,20]. *PbCSE9* is therefore probably involved in lignin biosynthesis of pear fruit.

### 3.6. The methylation Steps

S-adenosyl-L-Met-dependent O-methyltransferase (OMT) is widely present in plants and acts on Phe-derived substrates during the production of plant secondary compounds other than lignin. CCoAOMT and COMT are members of the OMT family, and they play an important role in the methylation step of the monolignol pathway [61].

**Figure 8:**
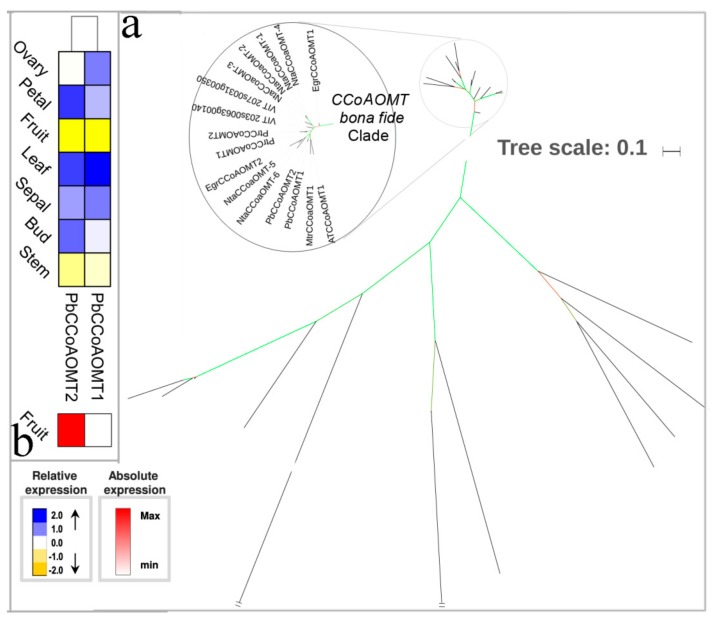
Phylogenetic tree and expression profiles of the CCoAOMT bona fide clade. (**a**) ML tree built with CCoAOMT bona fide enzymes from several species. (**b**) The expression profiles *P. bretschneideri CCoAOMT* bona fide genes. Subclade containing the bona fide genes is highlighted. The green color of the tree branches suggests strong node support (bootstrap support ≥ 95% and SH-aLRT ≥ 75%). The FPKM values of *PbCCoAOMT* genes in different tissues are indicated in Table S3. The gene accession number is indicated in Table S4, Figure S3. The tree scale is the number of amino acid substitution per site.

CCoAOMT may mainly act on the conversion of caffeyl-CoA to feruloyl-CoA in vitro, while in vivo it mainly catalyzes the SAM-dependent methylation of the phenolic hydroxyl group in positions three and five [14,62]. In many plants, functional studies of CCoAOMT have shown that they play an important role in the synthesis of G units. In the present study, we identified two *PbCCoAOMT* genes belong to the bona fide CCoAOMT (Figure 8a), the same number as in *P. trichocarpa* and *E. grandis*. *PbCCoAOMT1* was placed on the chromosome 17, while *PbCCoAOMT2* was placed on Scaffold (Table S2). The expression of *PbCCoAOMT1* was strongly expressed in the petal, and *PbCCoAOMT2* were preferentially and strongly expressed in the leaf. We also noted that *PbCCoAOMT2* showed a six-fold higher expression relative to *PbCCoAOMT1* in fruit (Figure 8b). Therefore, *PbCCoAOMT2* is the most likely candidate involved in lignin biosynthesis of pear fruit.

**Figure 9:**
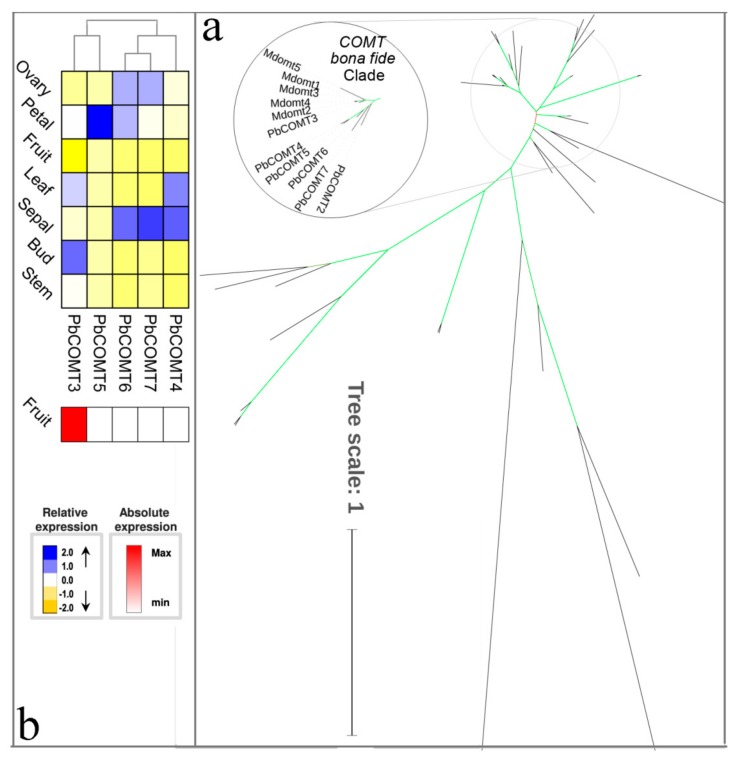
Phylogenetic tree and expression profiles of the COMT bona fide clade. (**a**) ML tree built with COMT bona fide enzymes from several species. (**b**) The expression profiles *P. bretschneideri COMT* bona fide genes. Subclade containing the bona fide genes is highlighted. The green color of the tree branches suggests strong node support (bootstrap support ≥ 95% and SH-aLRT ≥ 75%). The FPKM values of *PbCOMT* genes in different tissues are indicated in Table S3. The gene accession number is indicated in Table S4, Figure S4. The tree scale is the number of amino acid substitution per site.

COMT was initially described in angiosperms as a bifunctional enzyme methylating 5-hydroxyferulic acid and caffeic acid [14,62]. Recently, many *COMT* genes have been isolated and functionally studied in some plants, such as *P. trichocarpa* and *A. thaliana*. The transgenic studies suggested that the COMT gene was primary involved in S lignin biosynthesis in vitro [63]. In the present study, we identified five *PbCOMT* genes belonging to bona fide COMT. Compared to other bona fide clades with fewer *COMT* members (Figure 9a), the *P. bretschneideri COMT* gene contains five members that were derived from a different duplication mechanism. *PbCOMT4*, *PbCOMT5*, *PbCOMT6,* and *PbCOMT7* were all placed on a 20-Kb genomic region of chromosome 7 (Table S2), indicating that these genes were derived from tandem duplication events followed by functional divergence. *PbCOMT4*, *PbCOMT6,* and *PbCOMT7* showed similar expression patterns that were very different from that of *PbCOMT5*, presenting higher expression in sepal and low expression in the stem (Figure 9b). Additionally, the expression patterns of these *PbCOMT* genes revealed that *PbCOMT3* was expressed in the seven tissues examined, and the expression peaked in the bud and leaf. We also note that the amino acid residues described in this study related to the catalytic activity and chemical interactions of the COMT enzyme are completely conserved in PbCOMT3. Compared to the other *COMT* genes, *PbCOMT3* was also highly expressed in fruit (50-fold higher), so this gene was the most likely candidate for lignin biosynthesis of pear fruit.

### 3.7. The Two Last Reductive Steps

**Figure 10:**
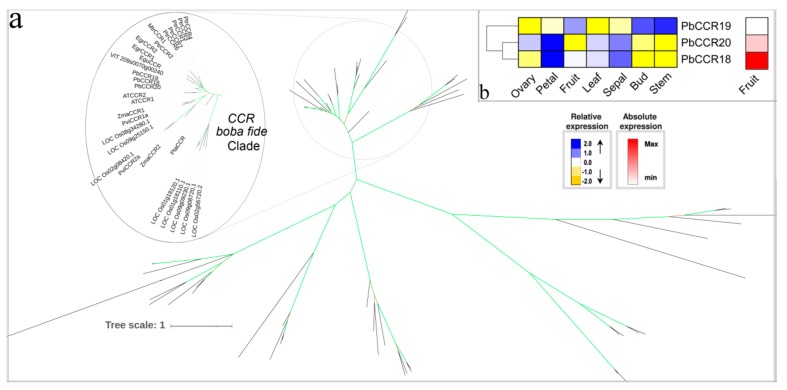
Phylogenetic tree and expression profiles of the CCR bona fide clade. (**a**) ML tree built with CCR bona fide enzymes from several species. (**b**) The expression profiles *P. bretschneideri CCR* bona fide genes. Subclade containing the bona fide genes is highlighted. The green color of the tree branches suggests strong node support (bootstrap support ≥ 95% and SH-aLRT ≥ 75%). The FPKM values of *PbCCR* genes in different tissues are indicated in Table S3. The gene accession number is indicated in Table S4, Figure S5. The tree scale is the number of amino acid substitution per site.

CCR catalyzes the reduction of cinnamoyl:CoA esters to their corresponding cinnamaldehydes, which is the first committed step of the monolignol biosynthesis. Purified enzymes from a number of species including *A. thaliana*, *E. grandis,* and *P. trichocarpa* were presented to be active towards sinapoyl-CoA, 5-hydroxyferuloyl-CoA, 4-coumaroyl-CoA, feruloyl-CoA, and caffeoyl-CoA [64,65]. Barakat et al. (2011) revealed and highlighted the bona fide CCR family by studying the CCR superfamily in land plants [66]. In the present study, we identified three PbCCR genes, and they all contained the CCR signature (NWYCY: Essential for its enzymatic activity) [67], including *PbCCR18*, *PbCCR19,* and *PbCCR20* (Figure 10a). In all clades, *AtCCR2* is mainly involved in defense mechanisms and respond biotic or abiotic stress, and its expression was poorly expressed during development; however, the *AtCCR1* gene plays an important role in developmental lignin biosynthesis [65]. Indeed, *PbCCR18*, *PbCCR19,* and *PbCCR20* were located on the same chromosome 17 derived from tandem duplication events followed by functional divergence. Both *PbCCR18* and *PbCCR20* showed similar expression patterns that that were very different from that of *PbCCR19*, presenting higher expression in the petal, followed by the sepal, and, finally, a low expression in the stem (Figure 10b). Additionally, *PbCCR18* was identified to be preferentially and highly expressed (15-fold higher) in fruit than other three genes—this being in accordance with a role for lignin biosynthesis of pear fruit.

**Figure 11:**
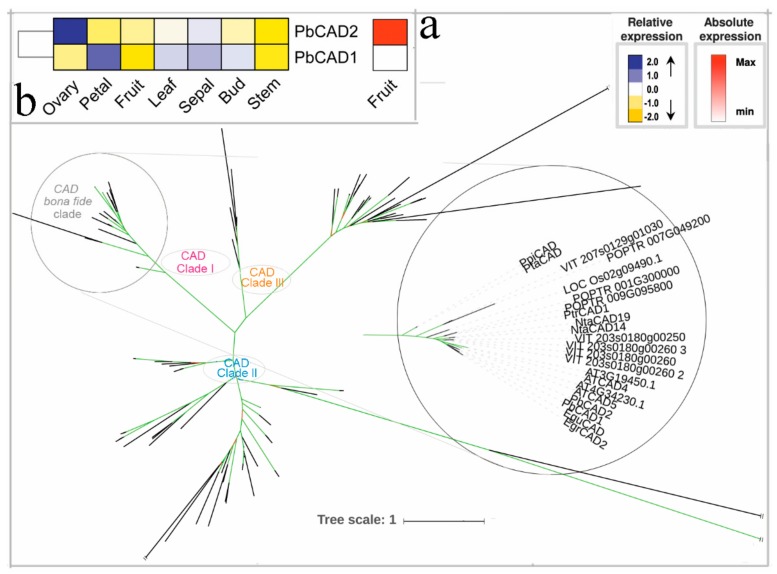
Phylogenetic tree and expression profiles of the CAD bona fide clade. (**a**) ML tree built with CAD bona fide enzymes from several species. (**b**) The expression profiles *P. bretschneideri CAD* bona fide genes. Subclade containing the bona fide genes is highlighted. The green color of the tree branches suggests strong node support (bootstrap support ≥ 95% and SH-aLRT ≥ 75%). The FPKM values of *PbCAD* genes in different tissues are indicated in Table S3. The gene accession number is indicated in Table S4, Figure S6. The tree scale is the number of amino acid substitution per site.

CAD is involved in the final step in the biosynthesis of monoligenol, which is capable of reducing cinnamaldehyde to alcohol. *CAD* and *CAD-like* genes have been identified in many plants, and they constitute large gene families such as *A. thaliana*, *E. grandis,* and *P. trichocarpa* [2,3,21]. Subsequent functional characterization confirms that different *CAD* gene family members have low homology and different affinities for various substrates and may have various physiological effects [68]. Previous studies have shown that the *CAD* gene family can be divided into three subfamilies during evolution, and there are certain differences between their evolutionary relationships and expression patterns [2,69,70,71]. As shown in Figure 11, clade I contains all bona fide *CAD* genes. Remarkably, in this clade I, *V. vinifera*, *N. tabacum*, *E. grandis,* and *A. thaliana* were represented by two *CAD* genes. *P. trichocarpa* (*PtrCAD1*) was the exception, with only one *CAD* gene involved in monolignol biosynthesis (Figure 11). In the present study, we found that *PbCAD1* and *PbCAD2* were placed on chromosomes 10 and chromosomes 14, respectively, a placement resulted from a segmental duplication events (Table S2). These two *PbCAD* genes show divergent expression patterns. *PbCAD2* highly expressed in the ovary, and *PbCAD1* preferentially expressed in the petal (Figure 11b). Remarkably, *PbCAD2* showed eight-fold higher expression than *PbCAD1* in fruit, indicating this gene was the most likely putative for lignin biosynthesis of pear fruit.

### 3.8. Hypothetical Pathways Involved in the Biosynthesis of Lignin in Pear Fruit

**Figure 12:**
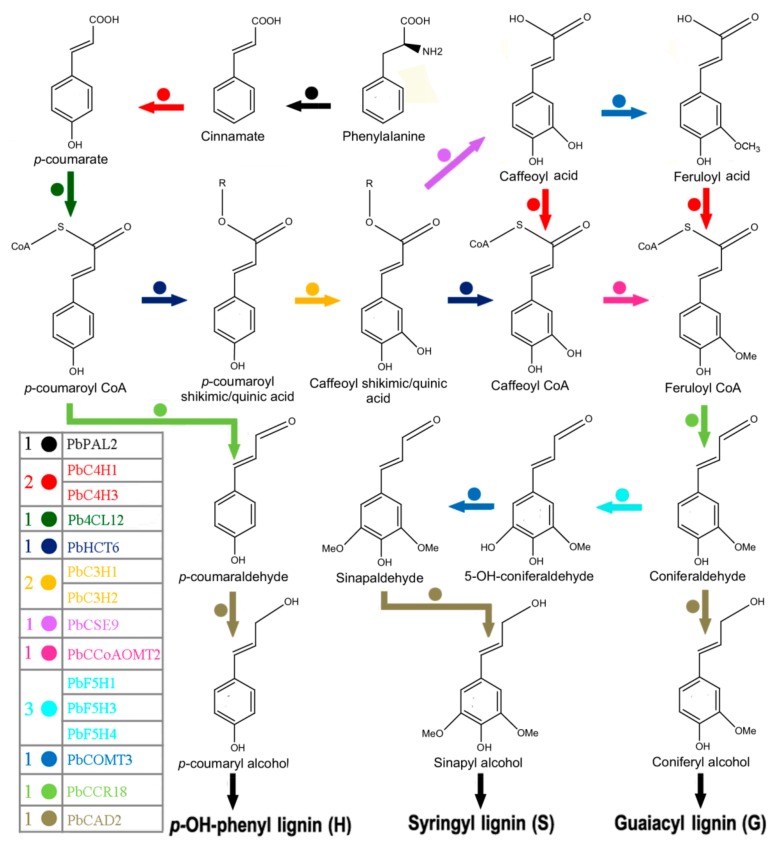
Hypothetical pathways involved in the biosynthesis of lignin in pear fruit. The biosynthetic pathway was adapted from previous findings [3,14,17,72]. The 15 *P. bretschneideri* genes encoding enzymes located in the bona fide clades constitute the core group of lignin synthesis in pear fruit.

Through a close combination of evolutionary analysis and expression patterns, we identified thirty-five bona fide clade members of eleven gene families in the *P. bretschneideri* genome, and then highlighted 15 as the most likely major genes involved in lignin synthesis of pear fruit. The core fruit lignification toolbox comprises *PbPAL2*, *PbC4H1*, *PbC4H3*, *PbF5H1*, *PbF5H3*, *PbF5H4*, *Pb4CL12*, *PbHCT6*, *PbCSE9*, *PbCCoAOMT2*, *PbCOMT3*, *PbCCR20,* and *PbCAD2* (Figure 12). To further obtain the expression profile of preferentially expressed and/or strongly expressed genes in pear fruit, we observed a unique expression pattern by using RNA sequencing data. In addition, we also noticed that some other genes are expressed in these eight tissues, such as *Pb4CL1*, *Pb4CL5*, *PbCOMT6*, *PbCOMT7,* and *PbHCT4*. Considering their expression patterns, we can speculate that these genes may be involved in the biosynthesis of other phenylpropanoid compounds other than the lignin metabolic pathway.

### 3.9. BiFC Assays

**Figure 13:**
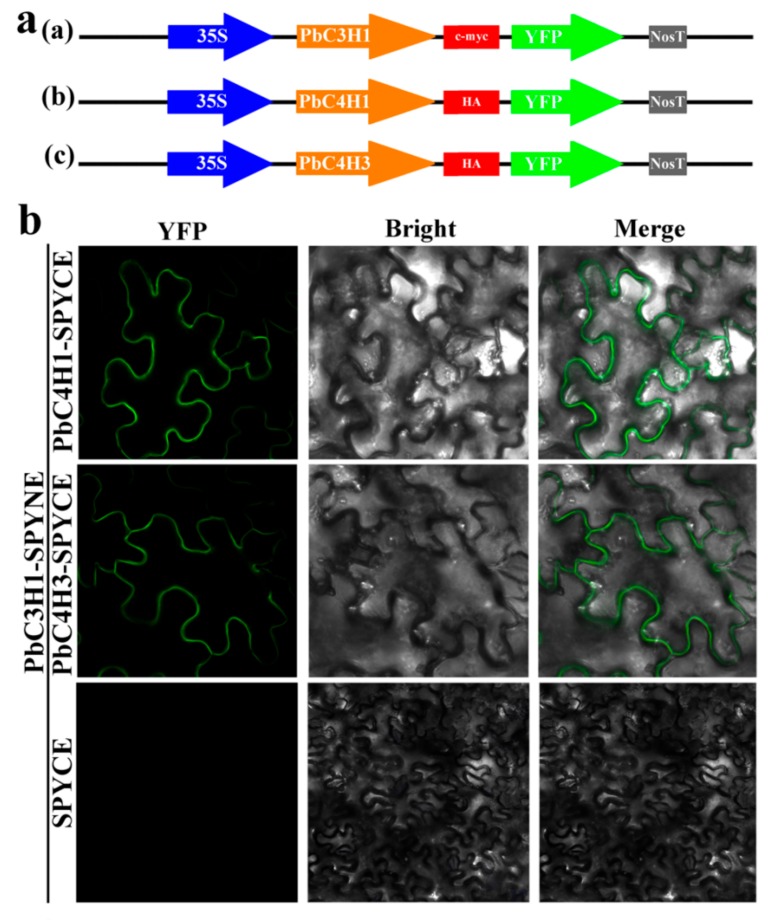
In vivo BiFC analysis of interaction between *PbC3H* and *PbC4H* co-expressed in *N. benthamiana* leaf cells. The coding regions of *PbC3H* and *PbC4H* were fused to the N-terminal and C-terminal halves of YFP, respectively. (**a**): Schematic representation of the 35S: PbC3H1-YFP, 35S: PbC4H1-YFP, and 35S: PbC4H3-YFP fusion constructs used for transient expression. (**b**)**:** A laser confocal microscope (Zeiss LSM700, Germany) was used to capture the fluorescence signals of the reconstituted YFP of the lower epidermal cells of leaves cut four days injection.

In the previous studies, Chen et al. (2011) found that *P. trichocarpa C3H3* (*PtrC3H3*) interacted with *PtrC4H1* and/or *PtrC4H2,* causing a significant increase in their catalytic activity and efficiency [73]. They may form heterodimers involved in monolignol biosynthesis [73]. To further understand such a molecular mechanisms in *P. bretschneideri*, we used the BiFC to explore interactions between the selected *P. bretschneideri C3H* and *C4H* genes. In the present study, *PbC3H1* was fused to the N-terminal fragment of YFP, while *PbC4H1* and *PbC4H3* were fused to the C-terminal fragment of YFP. A complete fluorescent YFP complex will form and be detected when the two tested proteins bind to each other. In the present study, we observed YFP fluorescence on the membrane when PbC3H1-YFPN was injected with PbC4H1-YFPC or PbC4H3-YFPC, but not with YFPC (Figure 13). Our data suggested that *P. bretschneideri C3H* and *C4H* might also interact with each other to regulate monolignol biosynthesis in *P. bretschneideri*, ultimately affecting stone cell content in pear fruits.

## 4. Conclusions

Stone cells, mainly composed of lignin, are important factors affecting the quality of pear fruit. In the present study, a systematic analysis of the evolutionary relationships and expression patterns of the lignin biosynthesis gene families in *P. bretschneideri* genome was carried out. Subsequently, we highlighted the evolutionary histories of these lignin biosynthesis gene families and those members who may be involved in the lignin synthesis of pear fruit. Our research provides a solid foundation for future functional studies and molecular breeding to improve pear fruit quality.

## Figures and Tables

**Table 1:** Identification of lignin biosynthesis genes.

Enzymes	Domains	*E*-Value
PAL *	PF00221/TIGR01226	1.00 × 10^−30^
C4H	PTHR19383:SF33	1.00 × 10^−30^
4CL	PTHR11968:SF43	1.00 × 10^−30^
HCT	PF02458	1.00 × 10^−30^
C3H	PTHR19383:SF44	1.00 × 10^−30^
CSE	PF03552	1.00 × 10^−30^
CCOAMT	PTHR10509	1.00 × 10^−30^
CCR **	PTHR10366:SF9	1.00 × 10^−24^
F5H	PTHR19383:SF46	1.00 × 10^−30^
COMT ***	PIRSF005739	1.00 × 10^−8^
CAD	PTHR11695:SF38	1.00 × 10^−30^

* According to previously published articles [30], the *E* value cutoff of 1.00 × 10^−24^ for CCR (cinnamoyl CoA reductase) gene identification was used. ** The identification of PAL (phenylalanine ammonia-lyase) genes involved two domains. *** According to previously published articles [30], we used *E* value cutoff of 1.00 × 10^−8^ for COMT gene identification.

## Supplementary Materials

The following are available online at https://www.mdpi.com/2218-273X/9/9/504/s1, Figure S1: Unrooted protein phylogenetic tree of the 4CL multigene family. Subclade containing the bona fide genes is highlighted. Green and red represent high and low bootstrap values respectively. Figure S2: Unrooted protein phylogenetic tree of the HCT/HCQ multigene family. Subclade containing the bona fide genes is highlighted. Green and red represent high and low bootstrap values respectively. Figure S3: Unrooted protein phylogenetic tree of the CCoAOMT multigene family. Subclade containing the bona fide genes is highlighted. Green and red represent high and low bootstrap values respectively. Figure S4: Unrooted protein phylogenetic tree of the COMT gene family. Subclade containing the bona fide genes is highlighted. Green and red represent high and low bootstrap values respectively. Figure S5: Unrooted protein phylogenetic tree of the CCR multigene family. Subclade containing the bona fide genes is highlighted. Green and red represent high and low bootstrap values respectively. Figure S6: Unrooted protein phylogenetic tree of the CAD multigene family. Subclade containing the bona fide genes is highlighted. Green and red represent high and low bootstrap values respectively. Table S1: Primers used in this study; Table S2: Identification of lignin toolbox in pear fruit; Table S3: The expression profiles of *P. bretschneideri* lignin toolbox bona fide genes; Table S4: Full-length protein sequences used to assemble the phylogenetic trees.
