# Supplementary figures and images for: Integrative Analysis of the Core Fruit Lignification Toolbox in Pear Reveals Targets for Fruit Quality Bioengineering
Source: Biomolecules. 2019 Sep 18;9(9):504. doi: 10.3390/biom9090504 (PMC6770946; doi:10.3390/biom9090504)

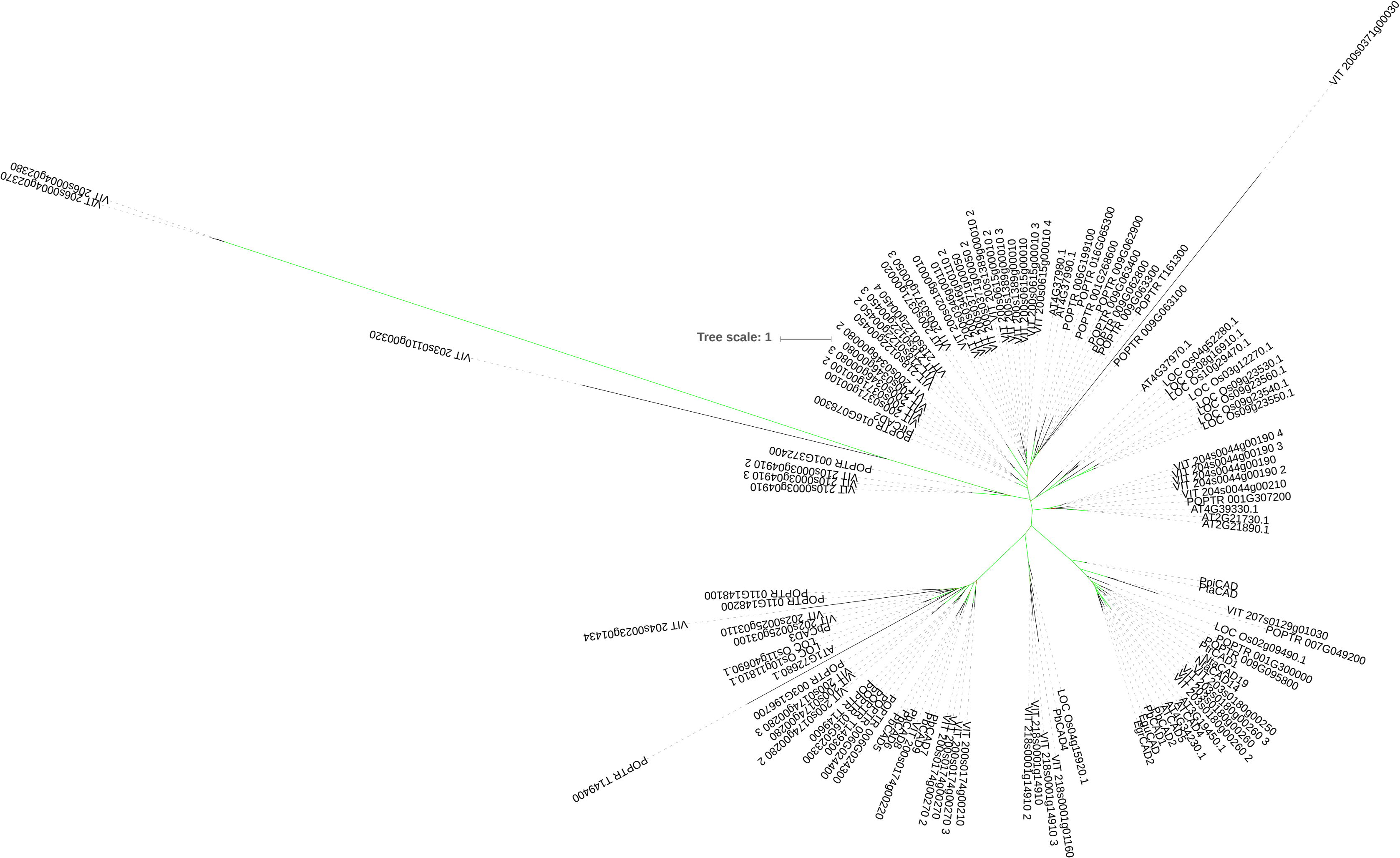

Supplement: Supplementary file 1 [file biomolecules-09-00504-s001.zip › Figure S6.pdf]

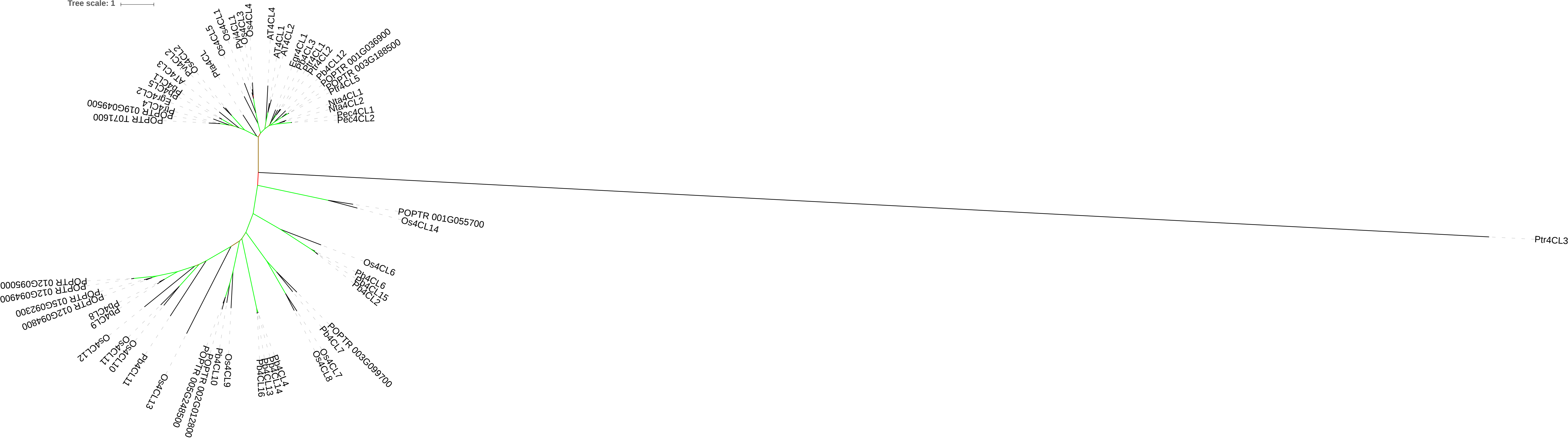

Supplement: Supplementary file 1 [file biomolecules-09-00504-s001.zip › Figure S1.pdf]

Tree scale: 1

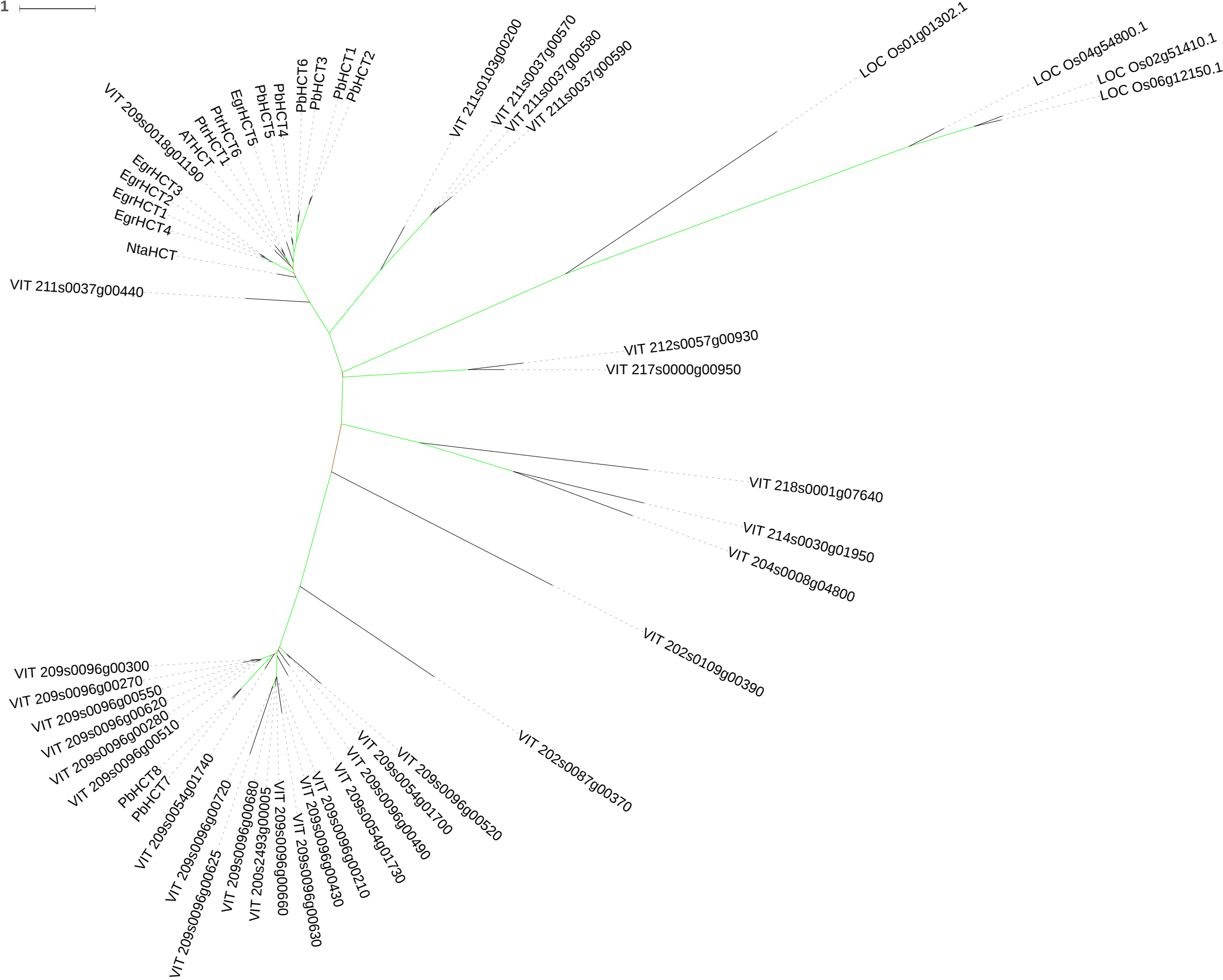

Supplement: Supplementary file 1 [file biomolecules-09-00504-s001.zip › Figure S2.pdf]

Tree scale: 0.1

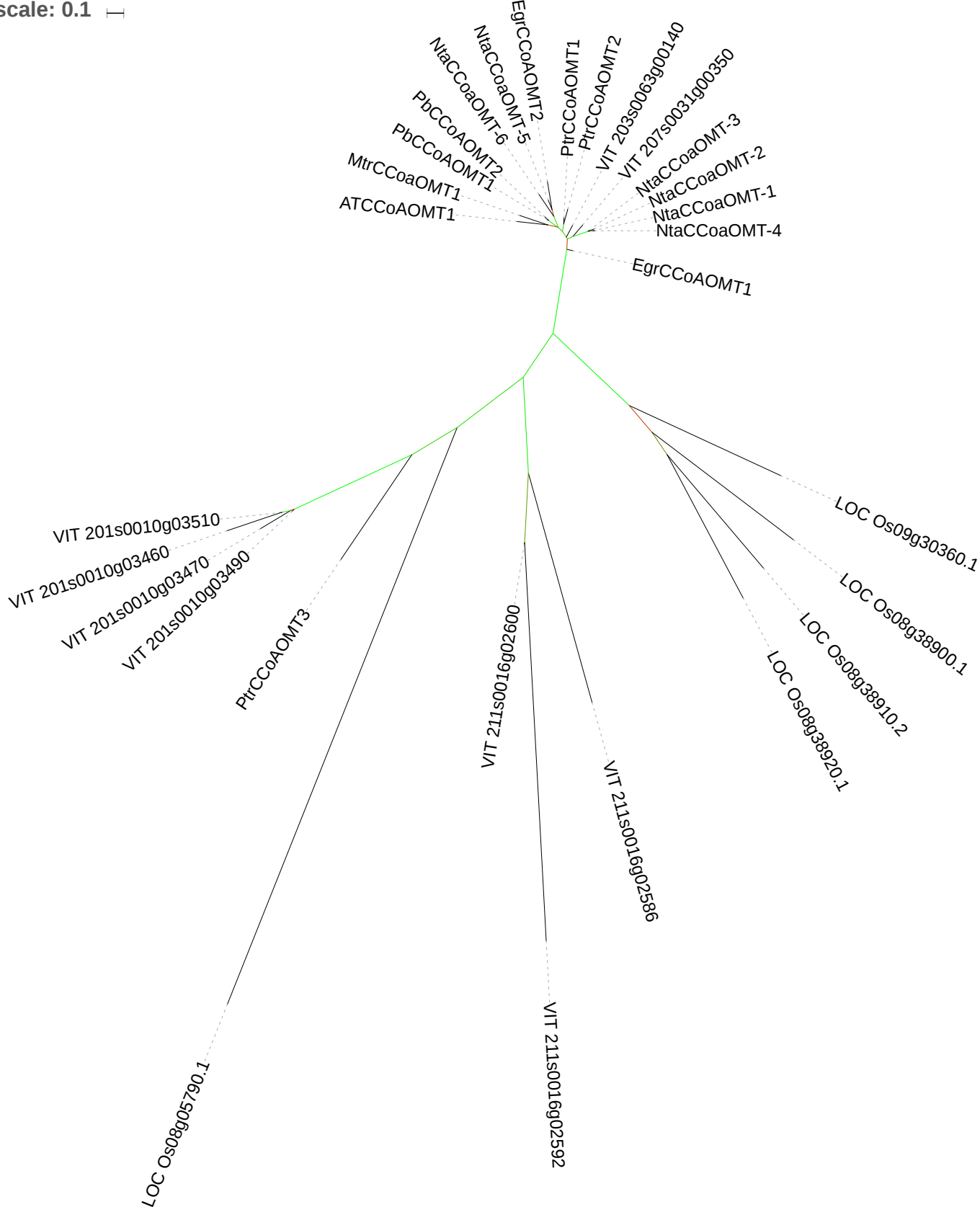

Supplement: Supplementary file 1 [file biomolecules-09-00504-s001.zip › Figure S3.pdf]

Tree scale: 1

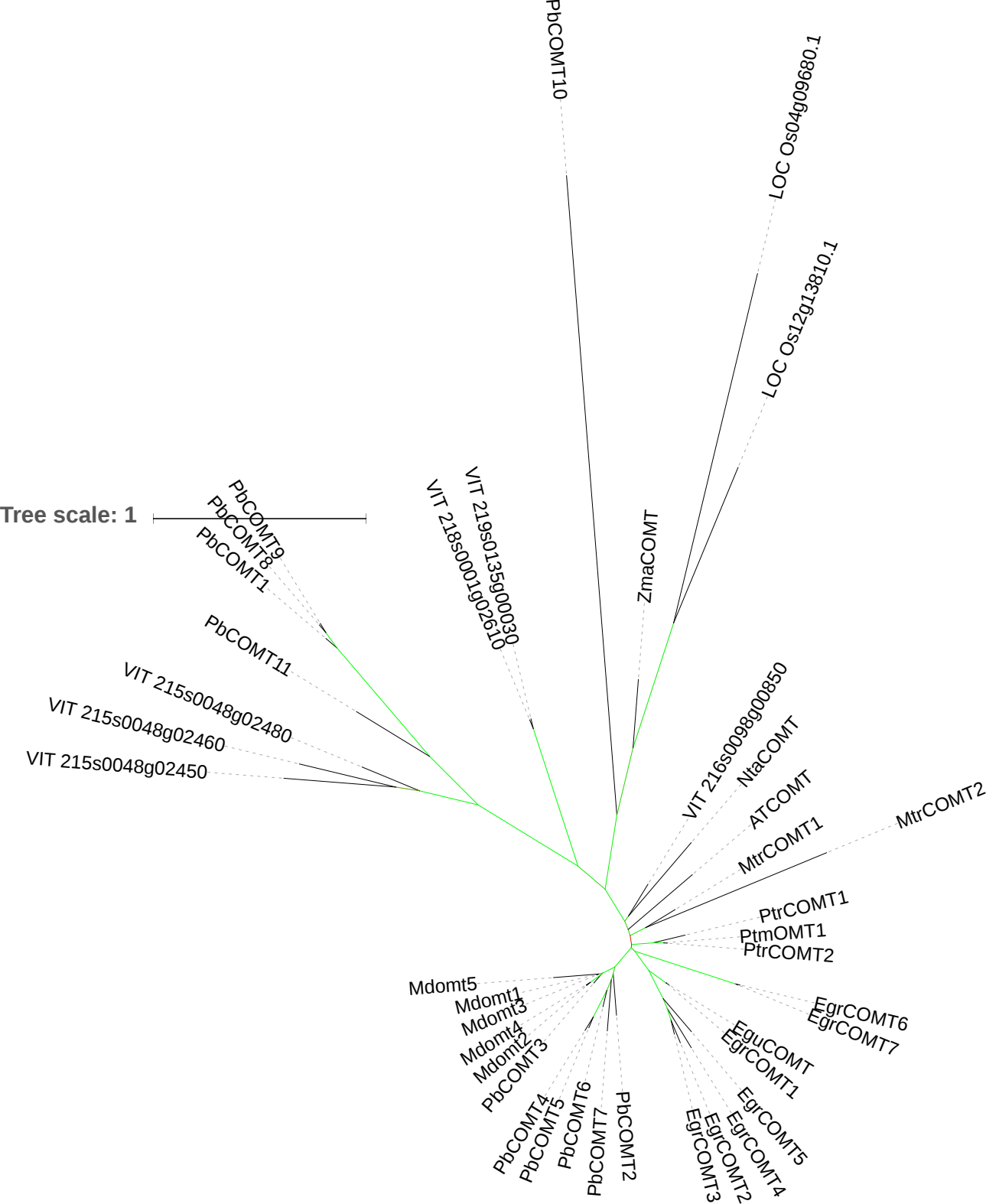

Supplement: Supplementary file 1 [file biomolecules-09-00504-s001.zip › Figure S4.pdf]

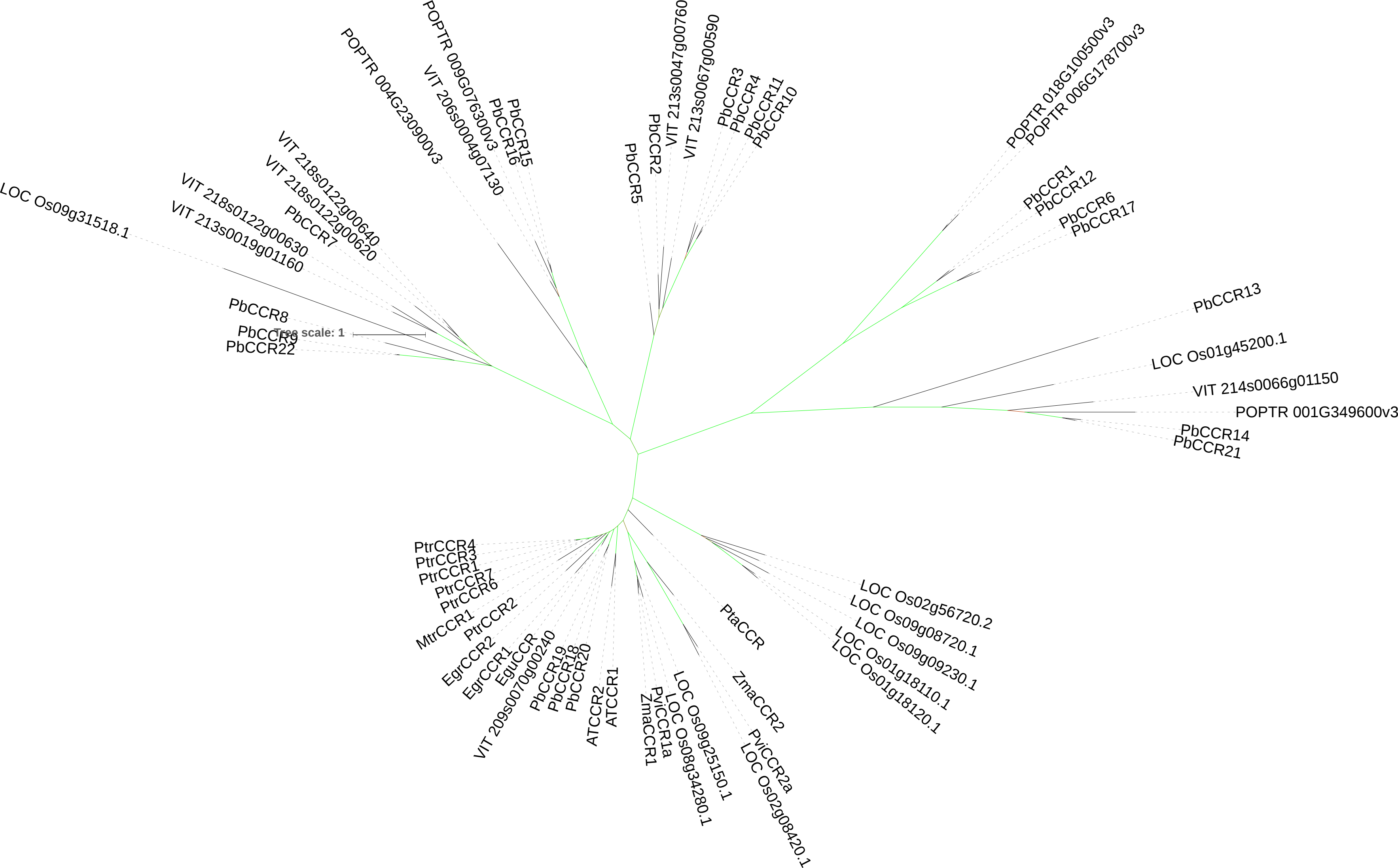

Supplement: Supplementary file 1 [file biomolecules-09-00504-s001.zip › Figure S5.pdf]
